# Key Informants Specify Core Elements of Peer Supports for Parents With Serious Mental Illness

**DOI:** 10.3389/fpsyt.2019.00106

**Published:** 2019-03-04

**Authors:** Joanne Nicholson, Anne Valentine

**Affiliations:** Institute for Behavioral Health and Lurie Institute for Disability Policy, The Heller School for Social Policy and Management, Brandeis University, Waltham, MA, United States

**Keywords:** parents with serious mental illness, peer supports, family-focused care, psychiatric services, program theory, evidence-based practice

## Abstract

**Background:** Researchers have documented the lack of evidence-based interventions for parents with serious mental illness (SMI). Given the prevalence of parenthood among individuals with SMI, the value placed on parenthood, and their diverse vulnerabilities, a robust, theoretically sound and empirically tested model of peer supports would likely provide a valuable complement to psychiatric services. In this paper, we lay the groundwork for a model of peer supports using a program theory development process and guided by stages of evidence-informed innovation outlined by the National Implementation Research Network.

**Methods:** This study employed a developmental design in the initial stage of a larger study of the development, implementation, and testing of peer supports to address three questions: (1) What needs of parents with SMI are particularly well-suited to peer supports? (2) What do peers have to offer parents? and (3) What is unique about peer supports for parents? A purposeful sampling strategy was used to recruit key informants (*n* = 22) familiar with peer supports, family-focused care and the experiences of families living with parental mental illness. Individual interviews were conducted face-to-face or on the telephone and a full-day group interview was conducted using a workshop format. Interview data were analyzed qualitatively to identify themes reflecting potential core program elements.

**Results:** Consistent themes drawn from data comprise four core program elements: engage, explore, plan, and access and advocate. These core activities are likely founded on practice principles that include a focus on families and their strengths, cultural sensitivity, and acknowledgment of the trauma experienced by many parents. The findings raised a number of challenges in contemplating peer supports for parents with SMI, including the need for ongoing support for peers.

**Discussion:** In developing this model, aspects of organizational context must be considered along with specification of the characteristics of parents with SMI suited to this approach and the attributes of peers providing support. A fully-articulated model must include parallel theories of change for the workforce, as well as for participating parents, to support well-being in the context of peer relationships and the success of parents with SMI in family life.

## Introduction

Numerous researchers have documented the lack of targeted evidence-based interventions for parents with serious mental illnesses (e.g., schizophrenia, bipolar disorder, major depressive disorder) (1, 2). Effective psychiatric treatment is recommended, of course, dependent on a person's history, diagnosis, preferences and response. However, inattention to issues of importance to individuals with serious mental illnesses (SMI) who are parents, and the probable impact of unaddressed issues on a person's participation and progress in treatment, suggest the potential benefit of attending to a parent's circumstances and priorities, particularly if they are contributing to stress. Given the prevalence of parenthood among individuals with SMI (3, 4); the value placed on parenthood by these individuals (5, 6); and their diverse vulnerabilities (e.g., limited resources and supports) (7), a robust, theoretically sound, empirically tested model of peer supports for parents with SMI would likely provide a valuable complement to traditional psychiatric treatments. In this paper, we lay the groundwork for a model of peer supports for parents using a program theory development process (8) and guided by stages of evidence-informed innovation outlined by the National Implementation Research Network (NIRN) (9).

Peer-delivered service models have increased markedly over the past two decades and are now an integral component of the behavioral health care system in the USA (10, 11). Peer support is based on the idea that individuals with lived experience of mental health and/or substance use disorders who have made progress in recovery may be uniquely positioned to offer support, validation, and hope to others (12, 13). Within the field, peer support specialists are persons who identify as having mental health conditions who receive formal, skills-based training to deliver services in the behavioral health care system (14). Peer support specialists occupy a variety of roles in behavioral health care that vary by setting, credentialing requirements, and service model (6, 15). In the USA, states are authorized to bill Medicaid, the public insurer, for peer support services to assist individuals in recovery from mental illness and substance use disorders.

A growing body of literature supports the benefits of peer-delivered services in behavioral health care (16). Much of this literature suggests positive outcomes as demonstrated by increasing consumer engagement and retention in care, linking individuals to community-based support services, fostering hope, addressing social isolation, increasing activation and self-care, and reducing inpatient utilization of services (11, 13, 16–21). However, recent meta-analyses of peer-delivered services, particularly for individuals with SMI, provide a more tentative assessment of the empirical evidence and raise questions about the methodological rigor of published studies (18, 22, 23). Adequate categorization and clarity surrounding the role, objectives and performance of peers is often lacking (24–26). Theory-based program models of peer support with well-defined change mechanisms are needed (22, 25, 27), as is increased attention to implementation processes and barriers that might affect participation and anticipated outcomes (22, 25).

The task of specifying the core program elements and underlying processes of a model of peer supports for parents with SMI, along with clear specification of the role of parent peer specialists, is informed by review of the literature regarding peer supports in mental health in general (13, 20, 28, 29). Authors describe and link peer support processes to conceptual underpinnings. Chief among these, by definition, is the importance of lived experience with mental illness and within the system of services (13, 20, 29). The peer specialist's use of positive self-disclosure and the perception of commonalities shared with and by the patient or client contribute to relationship engagement and foster hope for change (13, 20, 29). The peer relationship is enhanced by the emotional engagement that develops in this work through trust, acceptance, understanding and empathy (13, 20, 29). Peer specialists have the ability to role model progress in recovery and coping. They may be more likely than clinical practitioners to take a strengths-based approach, rather than focusing on psychopathology, and facilitate access to social and practical support, both informal and formal (13, 20). The peer specialist role has been described as having benefit to the peer specialist as well as to the patient or client, via the assumption of the helper role (13, 29).

However, the possible emotional closeness between peer specialist and patient or client, in combination with the fact that the peer specialist role definition often lies somewhere between that of traditional mental health clinician and patient or client, may contribute to difficulties (29). The peer specialist may be vulnerable to over involvement, unsustainable boundaries and burnout (29), suggesting the need for ongoing training and support. The organizational or agency context (e.g., hospital in-patient setting, community mental health agency or health center) in which peer support is offered must be considered as well (29). Authors recommend that the process of creating peer positions should include the active involvement of non-peer staff, organizational leaders and people in recovery, especially those who may serve as champions in the effort (20).

This paper reports on the initial, preparatory and foundational stage of a multi-stage study to develop, implement and assess the effectiveness of peer supports for parents with SMI. It reports on work done together with diverse stakeholders in laying the groundwork for model development of peer supports targeting parents with SMI. The initial tasks include developing program theory linked with core elements and processes, and identifying essential qualities and characteristics of parent peer specialists. Research questions include: (1) What needs of parents with SMI are particularly well-suited to peer supports? (2) What do peers have to offer parents? and (3) What is unique about peer supports for parents (i.e., what distinguishes them from peer supports in general)? Findings from this initial exploration will inform the next stages of the project: first, further elaboration of the peer supports model for parents with SMI and, second, the installation, implementation and testing of the resultant innovative, empirically informed model.

## Methods

### Design

The study reported here employed a developmental design as the initial stage of the larger study of the development, installation, implementation and testing of parent peer supports. A developmental design has the purpose of helping develop an innovation, intervention or program (30). Feedback loops inform ongoing program, staff and organizational development. This approach is particularly relevant in situations in which programs or models are being adapted to new conditions, contexts or target populations (30), that is, the exploration and installation stages outlined in the NIRN framework for the implementation of evidence-based programs and other innovation (9). The larger study methods combine three iterative approaches to program theory building (8): (a) a review of the academic and gray literature (deductive approach); (b) interviews with key informants (articulating stakeholder mental models); and (c) Implementation Team meetings as the potential program model is considered for installation at the community-based agency (inductive approach). In this paper, we report findings from interviews with key informants. The extensive literature review, reported above only briefly, focused on the experiences and needs of parents with SMI and the development, implementation and testing of peer supports in general. Implementation Team meetings are underway as we prepare to install peer supports for parents with SMI in the community agency context.

### Key Informant Interviews: Articulating Stakeholder Mental Models

Mental models for programs reflect the ways in which stakeholders describe what a program would look like, how it would work, and how success would be determined, particularly as stakeholders consider the characteristics, needs and experiences of the target population (8). The selection of key informants is informed by the notion that engaging stakeholders provides access to expertise, facilitates the development of a shared understanding of the potential program and promotes empowerment, communication and uptake of the final result (8). Diverse perspectives are included to develop a more comprehensive program theory.

### Sample Recruitment

A purposeful sampling strategy was used to recruit initial key informants familiar with peer supports, family-focused care and the specific needs and challenges of families living with parental mental illnesses. A preliminary list of potential study participants was constructed by the investigators, chosen to reflect diverse, relevant perspectives including state agency policymakers and funders, provider agency managers and staff, experienced peer specialists, parents with SMI, program developers and researchers. Additional informants were added to the sample as they were identified by study participants. Potential participants were sent a recruitment email informing them of the study and inviting their participation in an interview to discuss peer supports for parents with SMI, including relevant factors to consider in developing the proposed model and ideas about how to implement, sustain and assess the effectiveness of the model. All invitees agreed to participate (*n* = 22). The investigators were included as participants given the co-production nature of the project and their partnership role in facilitating key informant engagement and contributing to the design of the ultimate parent peer supports model (8, 30).

### Interview Procedures

Interviews were conducted by one of the investigators, experienced clinical researchers, either face-to-face or on the telephone, at the convenience of the participants, between March 2016 and July 2017. A study overview including the interview items was emailed to participants prior to the scheduled session. The individual interviews lasted 1–2 h. One full-day group interview was conducted using a workshop format with expert stakeholders to draw out rich perspectives on key elements of program theory in an iterative, face-to-face manner (8). At the beginning of each session, informed consent was obtained and participants completed a brief background survey (e.g., age, gender, race/ethnicity, education, training/discipline, organizational affiliation, and years in their current role). For those individuals interviewed by telephone, informed consent forms were emailed in advance, signed and returned by the participant. Study procedures were reviewed and approved by the state agency research review committee and the institutional review boards of the investigators' universities.

The investigators employed a semi-structured interview protocol informed by a series of open-ended questions as suggested in the NIRN Exploration Stage of innovation development (9). The questions were used to orient conversation. By way of introducing the topic of peer supports for parents, the interview began by asking participants for their perspectives on the needs of parents with SMI. Follow-up questions elicited information on current programs and practices, potential resources for the proposed parent peer supports, anticipated implementation and sustainability challenges, and additional stakeholders to contact. The interview concluded with an open-ended question asking whether the participant had anything else to add. The investigators began reviewing data as they became available, so that earlier interviews informed later interviews as knowledge was obtained. Verbatim notes were taken during all interviews, reviewed and transcribed by the study investigators using standard word processing software, and de-identified to maintain the participants' privacy. In addition, for the workshop session, extensive notes were made on easel sheets along with verbatim notes to facilitate group discussion. These were transcribed as well.

### Analysis

A framework approach to the interviews was employed, given that a starting set of issues to investigate was identified prior to data collection. These included recommended considerations in the NIRN exploration stage of innovation related to implementation context, such as the identification of essential resources, potential barriers, and lessons learned from prior initiatives (9). Open-ended interview questions were developed to provide for unstructured responses to these implementation topics as well as to allow for rich responses from study participants regarding the specific needs and experiences of parents with SMI (31, 32). While the overall framework of the interviews as focusing on developing and implementing peer supports was identified in advance, themes related to essential elements and considerations for the development and implementation of peer supports for parents with SMI emerged from the data, in a grounded theory type of approach, as thematic coding progressed (33). The investigators worked independently initially, recording notes of themes that emerged from the data, as well as informed by the research objectives. They reviewed and updated themes based on the frequency of themes within a response and the emphasis granted certain themes by participants. Together, investigators revisited and updated themes and came to consensus regarding the codes and the language used to define them. Coding was facilitated through the use of Dedoose software (34). The investigators generated memos regarding impressions and emerging themes and relationships, and reviewed findings as they supported, diverged from or augmented prior knowledge.

Trustworthiness of the qualitative analysis process and findings was established in multiple ways (35). Transcripts were reviewed and coded independently, impressions compared, and differences reconciled to achieve complete agreement in all cases. Trustworthiness was further established through member checking (33). Preliminary findings were reviewed by independent stakeholders on the study's Implementation Team and actively working in the field.

## Results

### Participants

Massachusetts, the seventh smallest state in the US, is located in the Northeast region of the country. The state population is estimated at 6.93 million. The state's capital, Boston, is the most populous city; more than 80% of Massachusetts residents live within the greater Boston metropolitan area. Prevalence estimates suggest that more than a half a million adults in Massachusetts experience SMI; unmet need for care is high. While Massachusetts is lauded as among the best states in the nation regarding access to behavioral health care, many individuals with SMI and substance use disorders struggle to find services.

Twenty-two informants participated in the study. The majority of informants were female (77%), over 45 years of age (77%), and Caucasian (86%), reflecting the characteristics of the Massachusetts human services workforce. Most had a four-year university degree or more in education (81%). The education of the remaining participants ranged from some high school to some college, with two completing a high-school equivalency diploma or trade school. Just over one-third of the participants (36%) were in their current positions for a dozen years or more; two were unemployed outside the home. Just over half (59%) described themselves as being in practitioner roles (e.g., social work, rehabilitation counseling, clinical psychology); 23% were in policy and program administration positions; and 18% were in other roles (e.g., mental health advocate; parent at home). Informants represented community mental health and state human service agencies, and included community leaders from varied practice settings and academic researchers, as well as advocates and parents with SMI. Their familiarity with peer supports and as service providers and recipients was extensive. Their experiences varied from working as peer support specialists and educators, to directing and supervising peer and recovery services to receiving peer services as patients or clients. Several informants were funders and administrators of peer services. A significant number identified as individuals with lived experience of mental illness or as having family members with lived experience. All were parents. The notion of peer support work in the US implies that those who direct or supervise these programs are often peers themselves, with lived experience of mental illness and as service recipients. It would be incorrect to assume that only those parents unemployed outside the home had ever received services. Participants were asked to designate their primary role as they identified it at the time of the study, but were encouraged to draw from their range of experiences.

### Research Question #1: What Needs of Parents With SMI Are Well-Suited to Peer Supports?

The gaps in supports and resources for parents with SMI were frequently noted by key informant participants. In addition, parents with SMI may have questions or concerns that are specific to their situations or circumstances. Needs that are common among all parents may be exacerbated in situations in which parents live with mental illnesses.

#### Gaps in Supports and Resources

Many participants touched on the lack of personal or professional support that parents with SMI experience, the pronounced sense of not knowing with whom to talk, and the implications of this isolation. As one key informant noted, “Often parents say they have no one who they rely on.” Participants observed that because so many parents with SMI do not have a “non-threatening person with whom they can confide” they may compartmentalize their needs and fear disclosure about salient information including the “use of psychiatric medication” and “childcare challenges.” Participants suggested that among parents with SMI, fear of the child welfare system and child protective services is a significant source of stress that may exacerbate parental isolation. Such parents might avoid seeking services lest disclosure of their psychiatric diagnoses or challenges in childrearing result in their children being taken from them. As one participant suggested, “the child welfare system exists to protect children, but there has to be a way for parents who are struggling to reach out—without the fear of having their children removed [from their care].” Participants also pointed out that parental isolation and the lack of support give rise to doubts and fears about a common concern of parents with serious mental illness, that is, if and when to disclose their illness to their children and others.

#### Concerns Specific to Parents With SMI

Peers, given their own experiences, may be best suited to discussing a number of issues that specifically relate to family life and serious mental illness. Participants strongly endorsed the use of peer supports for parents who may be “contemplating having another child” as peers may have experience with balancing the demands of parenting multiple children and managing serious mental illness. Peers may have insight into complex or stressful family relationships, for example, and suggestions for parents who have complicated relationships with their own parents, yet must “rely on [them] for child support.”

Many participants noted the crucial support that a peer may provide in helping parents reframe their experiences, to “deconstruct stigma” as they make negative assumptions about the relationship between their own illness and their children's behavior, and to “normalize the day-to-day challenges of parenting” that exist independent of a psychiatric diagnosis. Parents may blame themselves and their illnesses as contributing to behavior in children that actually may be developmentally appropriate (e.g., temper tantrums in toddlers). According to one informant, “It's common to ascribe any difficulties in parenting to depression, but there are normal experiences and challenges of parenting that exist apart from major depression.” Further, parents with serious mental illness may need extra support in “the pragmatic steps necessary to get through the day,” for example, figuring out “how to get their kids to the bus on time in morning” or “how to get them enrolled in a good afterschool program.” In helping parents with serious mental illness navigate these day-to-day challenges, peers may suggest alternative strategies that help to change parents' perspectives from that of feeling overwhelmed by demands and “failing” to one of coping and succeeding in managing everyday family life.

Parents may benefit from peer supports for situations unique to them. As participants pointed out, parents who do not have custody of their children may have supervised visitation as required by child protective services or may hope for reunification with their children. Peers may have experience with child welfare and advice for navigating the system successfully. They may be preferred, willing and able to supervise parents' visits with their children. A key informant described, “This is complicated, but it's worth taking on. How can a parent interact with a child, particularly if they have limited contact?” Another informant suggested, “Parents may need support for grieving the loss of parenthood—for those who opt not to have children” and for those who have lost custody or contact. Parents with SMI may need help repairing and renewing bonds with children. For older parents, peers may be helpful in assisting them to reconnect with their adult children.

#### Generic Parenting Issues Magnified

Participants also remarked on the needs of parents with SMI that are generic to all parents. Concerns about housing, healthcare, and education were frequently cited. However, many suggested that even the very basic needs of vulnerable individuals take on a different quality and urgency for parents with SMI who are primary caretakers of minor children. As one informant noted, “Do I have enough money to pay my electric bill this month?” may mean something very different for individuals who are parents than for those individuals responsible only for themselves. For parents with SMI caring for children, failing to provide for the essentials of daily living may exacerbate their doubts about their parenting abilities, mentioned above, and draw the attention and concern of others.

### Research Question #2: What Do Peers Have to Offer Parents?

Peers may be sensitive to the importance of empowerment and the contribution of successful planning and problem-solving to feelings of self-efficacy and recovery. They may be well-suited to helping parents access and advocate for supports and resources for themselves and their families.

#### Plan

Peers can draw from their own experiences to help parents solve problems, envision change and make concrete plans to achieve their goals. “Peers workers facilitate peer-driven goal planning.” Because of their unique perspective as peers, they may be better able to partner with the parent to “go to a school meeting; go to a doctor's appointment; go to get a prom dress…help plan a [parent-child] visit, plan birthday parties; talk about court and steps to get children back.” They may have tested strategies, not only for managing day-to-day life but, specifically, household, money and time management. According to an informant, the peer can focus on “whatever it takes for families to be successful.” Peers may see “that there is more than one way forward” and appreciate that recovery is not a straightforward path; there will likely be set-backs and relapses along the way. Peers may be more inclined than non-peers to recognize that “It's about helping the client with self-determination, autonomy and self-advocacy” and that by succeeding in planning and problem-solving, parents begin to see and believe that change is possible and that they are capable of making it happen.

#### Access and Advocate

One informant indicated, “The role of peer support is to provide information and support as needed.” Parents may not know how to access resources, that is, “how to get adequate support” from friends or family or how to “navigate communication” across a network of professional providers. Peer specialists who are parents may be better acquainted with community resources useful to other parents and especially to those living with mental illness. Peer specialists may facilitate “referral to grassroots, community-based programs; in-home recovery services; and community-based supports” and may also be helpful in “talking with family members at the request of the client [parent]” to help the parent build natural supports. Advocacy is a key element of the process of accessing and building supports and resources. An informant pointed out, “A significant element of advocacy is integrated into the peer-based model of care. The [parent peer specialist] must understand this context, and the role of advocacy and networking with other providers serving families.”

### Research Question #3: What Is Unique About Peer Supports for Parents?

Informants suggested that peers could offer a unique and specialized approach to supporting parents with SMI in terms of their posture or approach to engaging with parents as well as the specific skills and expertise they employ to explore parents' strengths, resources and supports, and elicit motivation to make desired changes. Specific challenges were suggested that reflect both the benefits as well as the vulnerabilities inherent in the peer-parent relationship.

#### Engage

Participants indicated that peers may readily be able to engage with parents with SMI with greater authenticity than can traditional mental health clinicians. As one pointed out, peers create “relationships that feel normal, not clinical.” They can “get real with parents in a way traditional mental health workers can't.” The assumption is that peer relationships are based on the concept of mutuality, which “tends to make people more open.” Mutuality is sparked by the perception of similarities and sharing of lived experiences. Peer specialists are trained in the use of self-disclosure to enhance relationship-building. A parent who has lost contact with children, for example, may feel better supported by someone who can talk openly about feelings attached to similar experiences and provide examples of ways in which they coped. Informants recommended a life span approach to parenting, that is, providing peer supports to parents at all stages of family life.

Participants suggested the importance of cultural sensitivity. In addition to cultural variations in the framing of mental illness and treatment, for example, the ways in which parents talk about mental illness with their children or their approach to child behavior management may differ from culture to culture. The notions of authenticity, mutuality and cultural sensitivity led to discussion of the match and fit between parents and peers. Participants emphasized the importance of choice. As one informant who is a parent with SMI pointed out, “some pairs fit better than others….Either side should be able to decline the match or ask for a re-match…. [You] need to consider the parent's needs and the peer specialist's needs in making the match.” According to another informant, “Not everyone wants peer support. For some, it's not the right time.” The question of whether peer supports for parents must be provided by persons who are parents emerged and, if so, what aspects of parenting should be considered as criteria for the match (e.g., age of children, custodial or non-custodial status, etc.). And in weighing the criteria for fit, the felt connection between peer and parent may matter more than particular aspects of lived experience.

The notion of choice was echoed by participants who emphasized the importance of peers taking a non-judgmental approach, given that parents with SMI so often feel judged. The value of a non-judgmental approach plays out in working together with parents to explore their families' strengths and vulnerabilities, plan for themselves and their children, and deal with the consequences of their actions. According to one informant, “People have to be given choices, including potentially risky or harmful choices,” suggesting that parents may make choices that peers consider ill-advised. Another suggested, “Peer workers endorse the dignity of risk.” Parents with SMI are often viewed negatively, with little regard paid to their efforts or capacity to care for their children. A peer specialist may take a more “normalizing” approach focusing less on “clinical appraisal” to make “more room for exploration of feelings that are stigmatized in clinical settings.” Allowing thoughts and feelings to be expressed without fear of censure may reduce the level of blame or shame parents feel about their choices.

#### Explore

Peers may be especially helpful in assisting parents to identify strengths and reframe their capacity to make change in their lives. In the words of an informant, “You may be combatting a family's story or narrative. Parent peer specialists may be able to support the client around this better than others…can change the story and uplift the children.” By helping parents to “figure out the life they want to live…in contrast to the treatment world, which she describes as ‘so clinical',” the parent peer specialist instills hope for change. A parent informant described a particular peer specialist as bringing “creativity, fun and positivity to her work” that allowed the parent to explore and envision a more positive future. This helps parents “to become unstuck,” to participate in planning and to muster the energy and resources to achieve goals.

Peers may provide examples of success to parents with SMI, according to key informants. Peer specialists are often selected into their positions by virtue of the progress they have made in their own treatment and recovery. Parent peer specialists may offer examples and role model successful efforts to cope with the demands of parenting and illness management or negotiate more positive relationships with children and family members. They may draw from their own life experiences, not only to build relationships through perceived mutuality, but to provide examples of ways in which they themselves have coped or made changes that might work for the parent.

#### Lived Experience of Family Life May Convey Challenges

Peer specialist training may involve exposure to practice skills and approaches that are generally relevant to parents with SMI (e.g., shared decision-making, problem-solving, skills-building). The work of peers is further informed by their own family values and lived experience of family life, as well as their experiences with mental illnesses. Consequently, in drawing from and disclosing their family experiences, peers may become vulnerable in ways that differ from or are experienced more keenly than when providing more generic supports. Peer specialists may understand that “boundaries between peer and client are more permeable [than those between a traditional clinician and client]” and “boundaries and use of self-disclosure are [therefore] emphasized in…training.” The focus on family life in working with parents has the positive potential to provide rich opportunities for sharing, but carries the risk of reminding the peer of family experiences or memories, possibly traumatic, that were painful or unpleasant. Adding family experiences to the shared lived experience of mental illness and recovery not only multiplies the opportunities for mutuality, but increases the pool of potentially painful or unresolved issues for the peer as well as for the parent.

The peer specialist may also be inclined to extend themselves for parents in ways they might not for patients or clients who are not parents, particularly because the well-being of children may be involved. As one informant pointed out, “Peers, for example, shouldn't be driving people to appointments, unless it's in the service of their work…talking to the client, introducing them to a support group.” And, “Peers are not taxi drivers or junior counselors.” Another informant suggested, “Peers cannot be held responsible for client behaviors, nor should they be asked to do favors for clients, including holding money or assisting with medications.” These issues could become particularly salient and potentially confusing to the parent peer specialist when children are involved in the home, for example, when a parent is unable to drive a child to a doctor's appointment or has no food in the kitchen. However, as another informant concluded, “Sometimes people need to be taken care of; that's what staff do. Sometimes people need someone to be with them; that's what peers do.”

## Discussion

### Parents With SMI May be Helped by Peer Supports

Diverse informants, including parents themselves, agree that parents with SMI are often isolated, dealing with gaps in available supports and resources. They may be challenged by situations or circumstances related to parenting while managing mental illness, including very practical, day-to-day challenges of raising children, relationships with children across the life span, and issues other parents face but that are exacerbated by the challenges of navigating treatment and recovery. Peers offer special knowledge, drawn from personal feelings and their own experiences, that may serve as a resource to parents. What is unique about peer supports for parents is the potential for relationship building and the capacity for open discussion based on the sharing of the lived experience of managing mental illness, family relationships and parenthood, and the authenticity and mutuality all this inspires. Parent peer specialists potentially offer examples of success, serve as role models, reframe deficits and set-backs, normalize parenting experiences, and disclose information about themselves with purpose and intent. The lived experience that peers share also may include expertise in navigating the health care and social service systems as a parent with SMI. For example, they may be aware of community resources or problem-solving strategies that have worked for them.

### Potential Challenges

The findings raise a number of challenges in contemplating peer supports for parents with SMI. Some are broadly relevant to peer support and others are particular to the role of parent peers (e.g., boundaries within the parent-peer relationship, the judicial use of self-disclosure, the potential for raising the peer specialist's own concern for painful family issues, and the peer's values associated with families, parenting and children). To some extent, these challenges exist across the range of peer supports (i.e., substance abuse recovery coaches, peer supports in elder care), but because young children may be involved—either living in the home or not—a more cautious approach may be warranted.

Working with parents, especially parents of young children, underscores the need for explicit, ongoing training and perhaps a willingness to re-examine norms or standards of practice, particularly given the assumptions, values and range of feelings that often surround parenting and family life. For example, the idea of a peer's role in honoring parent choice was noted by several informants, the underlying notion being that peer specialists are there to honor and support the autonomy of their peers. In reality, this aspect of peer support is not terribly distinct from the approach of traditional mental health clinicians, in the sense that clinicians may not weigh-in directly and/or advise patients or clients specifically about everyday decision-making. However, honoring patient or client choice is essential to peer support, in part, because in supporting autonomy, peers support clients' wellness, as opposed to their illness, and their potential for growth and recovery (21). This may be the mechanism behind a peer's authentic use of self. It is certainly one of the most salient and important aspects of peer support. For parents whose lives may be heavily intertwined with people telling them what to do (e.g., partners, extended family members, child protective service workers), the dignity of choice, of having a support person who does not mandate or require a particular response seems nothing short of essential.

However, there may be instances when the question is not really about the autonomy or wellness of the parent but, rather, the implications of the parent's behavior or actions for a young child. This may present new challenges for peer support specialists; indeed, supporting a parent's autonomy to make less than ideal decisions may convey far-reaching implications. With respect to peer supports for parents, this suggests two related ideas. First, peer specialists working with parents in the home may experience more moments of dissonance or distress than they might otherwise in working with adults with no children or in settings outside the home. The peer specialist working in the home may have greater exposure to the consequences of a parent's decision-making (e.g., an empty refrigerator). Peer support specialists may benefit from targeted training, coaching and support from supervisors and peers, and from knowledge about specific supports and resources for the parents and families with whom they work, to deal with this increased exposure. The very opportunity for enhanced relationship-building conveyed by shared lived experience also may result in peers being aware of issues about which more traditional office-based clinicians have no knowledge.

Second, peer supports may not be right for everyone—the parent or the peer. Even well-trained peer specialists may not feel comfortable “intruding” in the parent's family life. Peer support specialists may be asked to interact with family members, school personnel and other professionals. This may be distinct from work undertaken by other peer support specialists. It may not be lived experienced of parenting, *per se*, that makes the work successful but, rather, the ability to tolerate ambivalence and utilize personal disclosure in a way the fosters a sense of possibility, regardless of family circumstance. This suggests that peers who work with parents may not necessarily have to be parents themselves. Similar issues have been raised in discussions of “peerness,” the fit and match of peer specialists and patients or clients (28, 36). Even individuals who are not parents themselves have lived experience of being parented and family life.

### Study Limitations

This was an exploratory study with a developmental design. While the number of participants was relatively small, they drew from considerable breadth and depth of experience—as professionals and as parents. The needs and experiences of parents with SMI identified by informants were consistent with those identified in previous research and in other countries. In subsequent stages of the study we propose to specify fully, implement and test a model of peer supports for parents with SMI that will be in many ways determined by the context in which it sits. Peer support models have developed in the USA to fit not only the regional or local service system context and need, but to fit the fiscal structure of the American health care system and reimbursement for services. Therefore, the study findings generally reflect parent/family and system characteristics and conditions known to Massachusetts, though the participants included a number of national experts from other locations. While a peer support model for parents with SMI may well have international application, given the similarities in the needs and experiences of parents across countries, aspects of the implementation context must be considered if the model is to be adapted and replicated in other sites.

## Conclusions and Implications for Future Practice and Research

Moving forward to inform a model of peer supports for parents, consistent themes drawn from data obtained to address the original research questions can be grouped into four core program elements: engage, explore, plan, and access and advocate. These core elements or categories of activity are likely founded on practice principles that include a focus on families and their strengths, cultural sensitivity, and acknowledgment of the trauma experienced by many parents living with mental illness, at home (e.g., when they lose custody of children) and in the system of services (e.g., if they are approached by others through the lens of negative attitudes and expectations). Further translation of core elements and practice principles into peer support activities, and the implementation and testing of the peer supports model for parents with SMI remain the focus of next steps in the multi-stage study. We anticipate that parent peer specialists will require additional training and coaching to supplement content covered in the state's mandatory training of peer support workers. The training and coaching materials will be targeted and relevant to the population of parents served. The content of these materials will be determined in consultation with key informants and other engaged stakeholders.

As noted in the introduction, a number of questions have emerged about the efficacy and impact of peer supports in behavioral health services. However, in thinking about peer supports for parents, the question isn't about whether peer support makes a difference, but under what circumstances and with whom (21). This question needs to be asked not only of the potential parent patient or client, but of the potential peer support specialist as well. Not everyone has an affinity for this work. This is a simple statement, yet one that needs to be explicitly addressed. Clearly, those who choose to provide peer supports to parents must be supported themselves (24), if their goals and the goals of the parents with whom they work are to be met.

Aspects of the organizational context must be considered along with specification of the characteristics of parents suited to this approach and the attributes of peer specialists providing support. The job description of peers working with parents with SMI must be well-specified, with adequate training, coaching and supports spelled-out and provided, to ensure that peer specialists are activating core program elements and processes, without putting their own well-being at risk. The role of peer specialists vis a vis the roles of others in the service provision setting must be clearly articulated, with organizational players prepared for and supported to promote productive collaboration and respect for the contributions of each. It is fair to assume that, amongst clinicians, there are individuals with experiences of mental illness and family life, though they may not disclose nor explicitly draw from their lived experience as do peer specialists. All staff members may require organizational leadership and support in taking a family-focused approach. A fully-articulated model of peer supports for parents with SMI, therefore, must include parallel theories of change for the workforce as well as for participating parents, to support well-being in the context of peer relationships and the success of parents in family life.

## Ethics Statement

This study was carried out in accordance with the recommendations of the Dartmouth College Committee for the Protection of Human Subjects (CPHS#00030199) and the Massachusetts Department of Mental Health Institutional Review Board (Protocol#2017-04) and approved by these committees. Written informed consent was obtained from all participants.

## Author Contributions

JN: designed the study; JN and AV: collected the data, performed analyses, interpreted results, prepared sections of the manuscript, and contributed to manuscript revision, read, and approved the submitted version.

### Conflict of Interest Statement

The authors declare that the research was conducted in the absence of any commercial or financial relationships that could be construed as a potential conflict of interest.
